# Global research trends on the relationship between RNA m^5^C modification and cancer progression: a comprehensive visualization and bibliometric analysis (1900−2024)

**DOI:** 10.52601/bpr.2025.250002

**Published:** 2026-04-30

**Authors:** Peipei Yao, Fei Chen, Nan Zhang, Hafiz Ullah, Xuecong Shi, Xinglong Zhong, Li Zhou

**Affiliations:** 1 Key Laboratory of Polar Environment Monitoring and Public Governance, Ministry of Education, Department of Immunology, Wuhan University Taikang Medical School (School of Basic Medical Sciences), Wuhan 430071, China; 2 Gomal Center of Biochemistry and Biotechnology, Gomal University, Dera Ismail Khan 29050, Pakistan; 3 Department of Stomatology, Wuhan University Hospital, Wuhan University, Wuhan 430072, China; 4 Jiangsu Hengrui Pharmaceuticals Co., Ltd, Lianyungang 222002, Jiangsu, China

**Keywords:** RNA m^5^C modification, Bibliometric, Cancer progression, CiteSpace, VOSviewer

## Abstract

The m^5^C modification is one of the widely occurring modifications on RNA. In recent years, m^5^C modification on RNA has increasingly become a focal point in cancer research. Nevertheless, investigating scientific and quantitative research on the publication trends in this field can help us understand the research background and emerging hotspots, providing insights for targeting RNA m^5^C sites in cancer therapy. Co-occurrence analyses and visualizations, including authorship, keywords, genes and diseases, were performed using VOSviewer. CiteSpace was used to identify bursting institutions, keywords and references. R packages, including clusterProfiler, enrichplot and ggplot2, were used to visualize the enrichment results of GO and KEGG. The top contributors to this field were the United States and China, and the journals with the most publications were *Frontiers in Genetics* and *Frontiers in Oncology*. The most common keyword was “m^5^C methylation” and the most related genes were “NSUN2”, “AKT1” and “METTL3”. This study conducted a bibliometric analysis covering the development process of RNA m^5^C modification in cancer progression, identifying the countries, institutions, authors, journals, and publications in this field. Additionally, we found that the genes most closely associated with m^5^C are likely to play a significant role in the process of viral oncogenesis. These findings provide a comprehensive overview of RNA m^5^C modification during cancer research and insights into RNA m^5^C-tageted cancer therapy.

## INTRODUCTION

Currently, over 100 chemical modifications have been identified in cellular RNA, with methylation modifications playing a significant role in these processes. These modifications regulate various biological functions by modulating RNA splicing, translation, transport, and stability (Cui *et al.* 2022; Li *et al.* 2024a). In eukaryotic mRNA, the primary types of methylation modifications include inosine, pseudouridine (Ψ), N^6^-methyladenosine (m^6^A), N^1^-methyladenosine (m^1^A), N^3^-methylcytidine (m^3^C), and 5-methylcytosine (m^5^C) (Li *et al.* 2024a; Roundtree *et al.* 2017). Among these, m^6^A modification is the predominant form of methylation in human mRNA and has been extensively studied (Cui *et al.* 2022; Huang *et al.* 2020). However, in recent years, m^5^C modification has emerged as a significant focus of research (Trixl and Lusser 2019).

The m^5^C modification on RNA is derived from an active methyl donor, typically S-adenosylmethionine (SAM), which is added to the carbon-5 position of the cytosine base in RNA to form the m^5^C modification (Walbott *et al.* 2007). This modification is widely present across various types of RNA, including transfer RNA (tRNA), ribosomal RNA (rRNA), long non-coding RNA (lncRNA), small nuclear RNA (snRNA), microRNA (miRNA), and enhancer RNA (eRNA), with the highest concentrations observed in the tRNAs and rRNAs of eukaryotes (Cantara *et al.* 2011; Song *et al.* 2022). The m^5^C modification is introduced site-specifically by methyltransferases, such as the NOP2/Sun domain (NSUN) family 1-7 and DNA methyltransferase 2 (DNMT2) (Chi and Delgado-Olguín 2013; Song *et al.* 2022) and is recognized by binding proteins such as the Aly/REF export factor (ALYREF) and Y-box binding protein 1 (YBX1), which subsequently trigger downstream regulatory reactions (Chen *et al.* 2019; Yang *et al.* 2017). Additionally, m^5^C can be demethylated by eraser proteins, including Ten-eleven translocation methylcytosine dioxygenases (TETs) (Lio *et al.* 2019; Wojciechowski *et al.* 2014; Yang *et al.* 2022). With advancements in sequencing technology, the RNA modification landscapes can now be comprehensively identified within the transcriptome. Recent progress in m^5^C detection technologies, including single-nucleotide resolution sequencing methods (RNA-BisSeq, TAWO-seq, AZA-IP-seq, Nanopore-seq, and miCLIP-seq) and antibody-based sequencing (m^5^C-RIP-seq), has facilitated in-depth research into m^5^C modifications (Chen *et al.* 2021; Cui *et al.* 2017; Gu *et al.* 2023; Hussain *et al.* 2013).

The m^5^C modification on RNA can significantly influence various biological processes, including cell proliferation, differentiation, migration, and apoptosis (Li *et al.* 2024a). In recent years, m^5^C modification has emerged as a prominent topic in cancer research. By affecting RNA maturation, metabolism, translation, and degradation, m^5^C modification plays a crucial role in cancer occurrence, metastasis, development, drug resistance, and recurrence (Gu *et al.* 2023; Tang *et al.* 2023). Liu found that NSUN2 promotes the m^5^C modification of transcription factor E2F1 mRNA in ovarian cancer cells, thereby enhancing its stability. Furthermore, high expression levels of NSUN2 and YBX1 are associated with prognoses for ovarian cancer patients (Liu *et al.* 2024b). NSUN2 can mediate the m^5^C modification of QSOX1 and regulate mRNA translation in a YBX1-dependent manner, playing a vital role in the prognosis and treatment of patients with non-small cell lung cancer (Wang *et al.* 2023b). In 2005, the research team led by Kariko demonstrated that the application of m^5^C modification in mRNA therapy can effectively reduce the Toll-like receptor (TLR)-mediated immune response induced by mRNA in cancer treatment (Karikó *et al.* 2005). This finding underscores the association between m^5^C modification on RNA and cancer progression, providing significant insights for the development of new therapeutic targets. Additionally, interesting observations have demonstrated that viral infections regulate the expression of m^5^C methyltransferases (Feng *et al.* 2023; Li *et al.* 2024b). Understanding the regulation of m^5^C methyltransferases during viral infections is crucial for elucidating the molecular mechanisms by which oncogenic viruses affect methylation modifications and tumor development. Consequently, this topic has increasingly become a focal point of research.

However, over the past two decades, the rapid increase in studies on this topic has resulted in an information overload, making it challenging for scholars to discern research trends and the latest hotspots. Therefore, an in-depth quantitative analysis is essential to fully understand the current research focus and to guide future research directions.

Traditional review articles summarized advancements in specific research fields. However, they may lack objective and comprehensive perspectives due to the subjective viewpoints and visions of the authors. Conversely, research articles focus on specific issues or particular research topics for in-depth analysis, yet they often fail to encompass the broader scope of the entire field, resulting in a lack of a comprehensive overview. Bibliometric, a branch of information science, quantitatively and qualitatively analyzes literature systems and bibliometric characteristics. This method facilitates the quantitative study of the distribution, relationships, and clustering of research topics, and has emerged as a popular technique for evaluating the credibility, quality, and impact of academic work (Ma *et al.* 2020, 2021). Furthermore, bibliometric analysis can help predict future research trends in specific fields, addressing the limitations of both review articles and research articles, thereby providing a comprehensive overview of the characteristics within a given field. Although bibliometric analysis has been extensively applied across various research domains, it has not been thoroughly investigated in the context of the relationship between m^5^C modification on RNA and cancer progression.

To enhance our understanding of the role of RNA m^5^C modification in cancer and to identify the frontiers and hotspots within this field, we summarized relevant scientific research findings from January 1900 to January 2024. By employing bibliometric methods, we identified 216 highly relevant academic articles, analyzing the contributions and impacts of the authors, countries/regions, institutions, disciplines, and journals associated with these works. As of the publication date of this article, the most recent publications on this topic align with the trends we have summarized, demonstrating the accuracy and referential value of our research (Chen *et al.* 2024b; Gu *et al.* 2024; Nulali *et al.* 2024). Furthermore, we focused on research hotspots, keywords, related gene enrichment analysis, and associated diseases. Our research integrates current findings in this field and outlines future development trends, offering new insights for cancer treatment and prognosis for researchers and clinicians, thereby providing significant reference value.

## RESULTS

### Overview of publications and time trends

**Figure 1:**
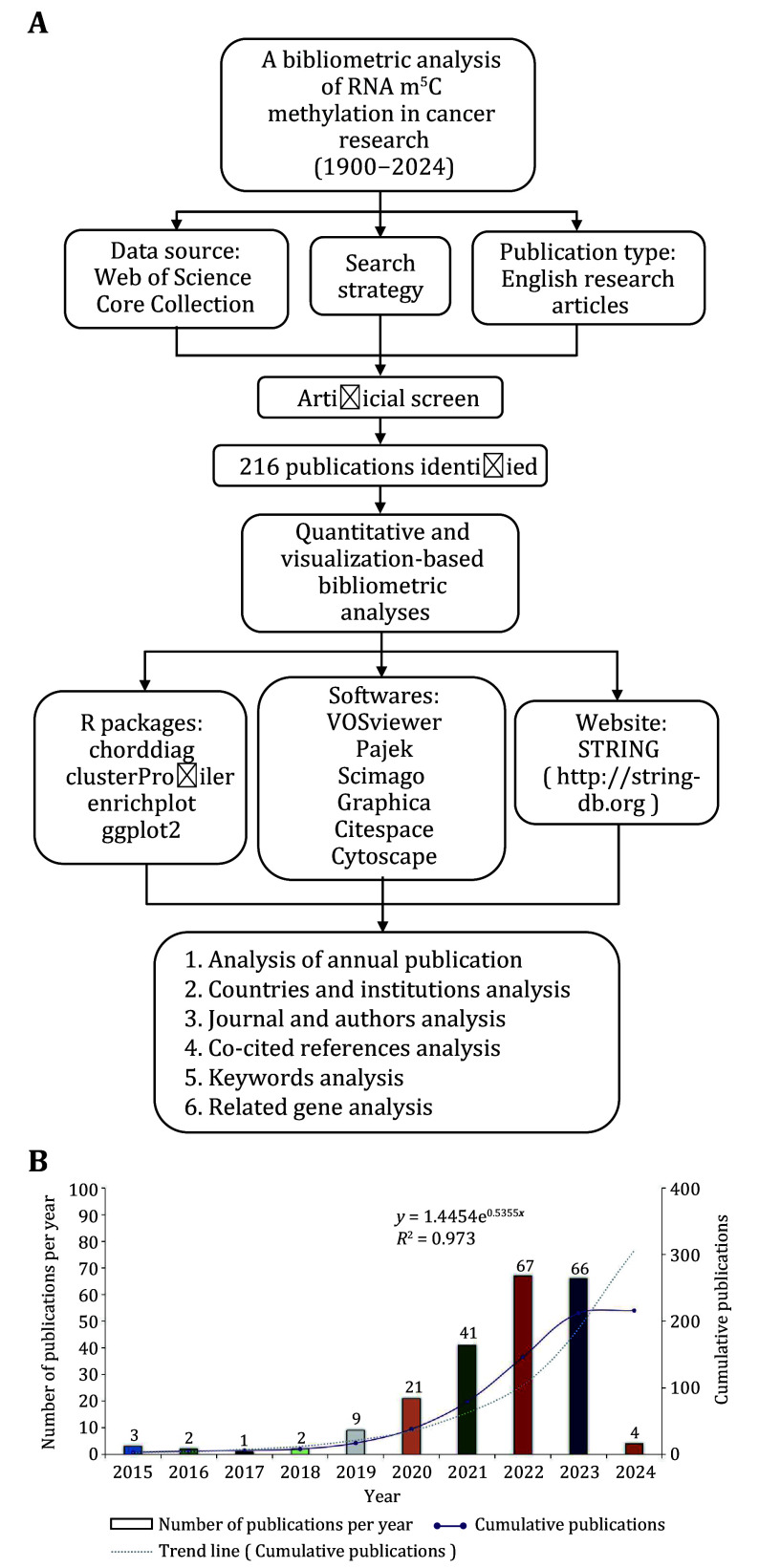
**A** Flowchart about the data collection, search strategy and methods of this study. **B** Number of publications per year and the cumulative number from Jan 1900 to Jan 2024

In this study, we identified a total of 216 articles on “the impact of m^5^C modification on RNA in cancer progression” from the Web of Science Core Collection database (Fig. 1A). Among these, 165 are research articles (76.4%), while 51 are reviews (23.6%), indicating a strong preference for original research over review articles in this field. We observed that the number of publications on this topic has increased from three articles in 2015 to 66 articles in 2023, with new studies being published each year. The cumulative number of publications has shown an upward trend over time, reflecting a growing interest and activity in this area. To further analyze this trend, we employed an exponential function, *y* = 1.4454e^0.5355*x*^ (*R*^2^ = 0.973), in which *x* represents the first year and *y* denotes the cumulative number of articles, to model the cumulative publication trend. This model exhibited a well-fitted curve, as illustrated in Fig. 1B. The steady annual increase in publications in this field can be attributed to factors such as enhanced accessibility and knowledge sharing facilitated by open-access journals. Research on “the impact of m^5^C modification on RNA in cancer progression” has become a prominent focus within the discipline. By considering various factors comprehensively, we can predict future research and publication trends in this area. As of January 23, four related articles have already been published in 2024 (Fig. 1B).

### Analysis of countries/regions and institutions

**Figure 2:**
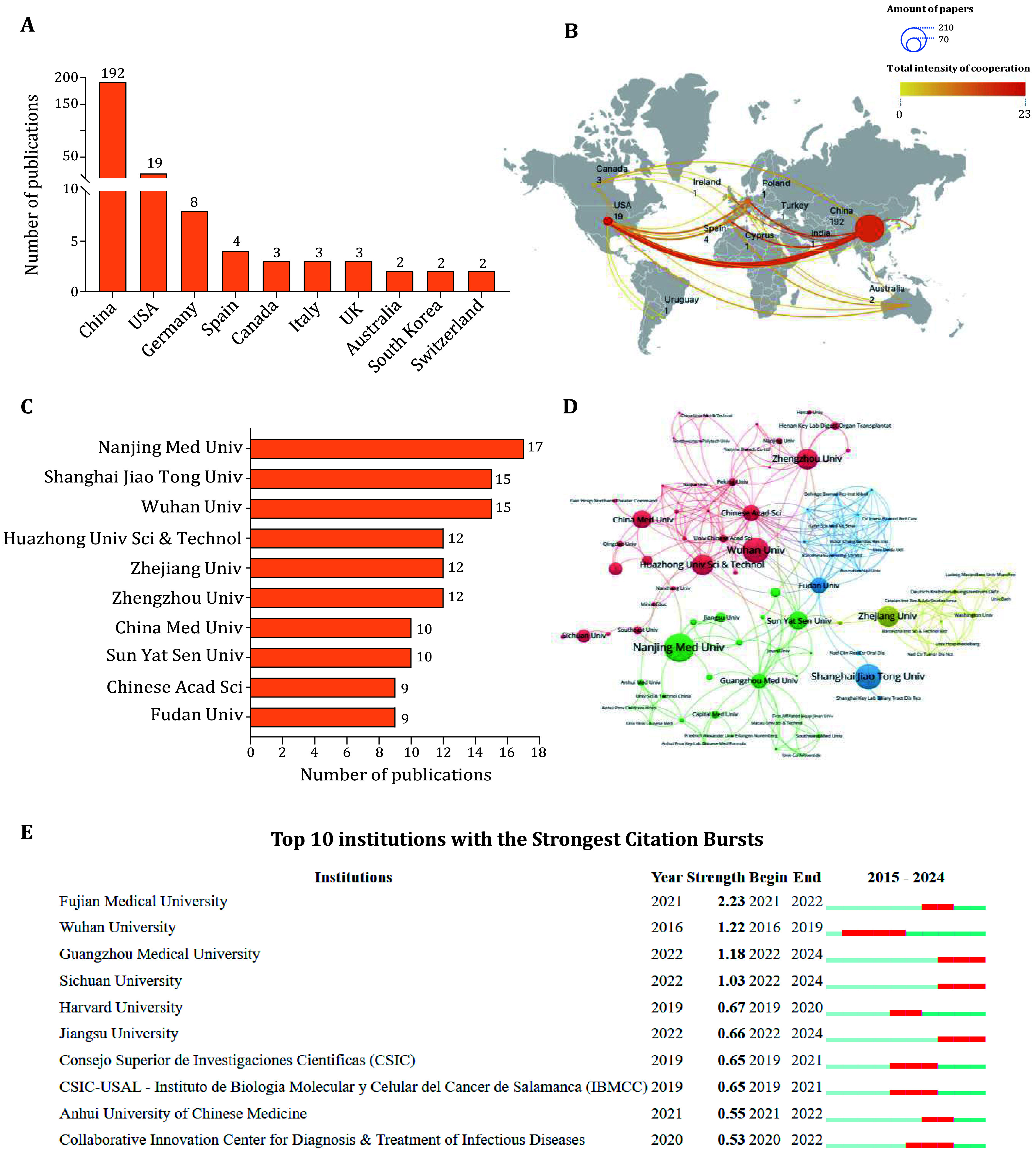
Country and institution analysis. **A** Number of publications in major countries. **B** Global map showing the cooperation network among countries. **C** Number of publications in major institutions. **D** Institutions cooperation network map. **E** The 10 institutions with the strongest citation bursts

We utilized the chorddiag R package and VOSviewer to visualize the contributions of various countries and regions in this field. The findings reveal that China leads with 192 articles, followed by the USA with 19 articles, Germany with 8, Spain with 4, Canada with 3, Australia with 2, and France with 1 (Fig. 2A). Furthermore, we examined the collaborative relationships among countries and regions, identifying that the most frequent collaborations occur between China and the USA (with a relative collaboration strength score of 9), as well as between the USA and Germany and the USA and Spain (Fig. 2B, supplementary Fig. S1A). Our data indicate that China and the USA are currently at the forefront of this field, demonstrating a close collaborative relationship. Although the USA ranks second in terms of publication count, it exhibits a strong willingness to collaborate, maintaining cooperative ties with multiple countries.

Subsequently, we visualized the contributing institutions within this field. Our screening of 216 articles revealed the involvement of 256 institutions, with the top 10 contributors all located in China (Fig. 2C). Nanjing Medical University emerged as the most productive institution, publishing 17 articles, followed by Shanghai Jiao Tong University and Wuhan University, each contributing 15 publications. This indicates that China plays a significant role in research within this domain. We also selected the top 100 collaborating institutions to visualize their cooperative relationships (Fig. 2D). Notably, the Chinese Academy of Sciences demonstrated the strongest willingness to collaborate, with the closest partnerships observed between the Chinese Academy of Sciences and the University of Chinese Academy of Sciences, Zhengzhou University and Henan Key Laboratory of Digestive Organ Transplantation, as well as Wuhan University and Huazhong University of Science and Technology. Each of these pairs of closely collaborating institutions is situated in the same region of China, suggesting that geographical proximity facilitates collaboration and enhances the willingness of nearby institutions to work together.

Furthermore, we conducted an analysis of institutions exhibiting bursts of activity in this field from 2015 to 2024 using CiteSpace. We identified the top ten institutions with significant bursts and their corresponding periods (Fig. 2E). The data indicates that Fujian Medical University experienced a notable increase in publications from 2021 to 2022, with a burst strength of 2.23. Wuhan University demonstrated the longest burst period, reflecting sustained productivity in this area of research. Other institutions that have experienced publication surges in recent years include Guangzhou Medical University, Sichuan University, and Jiangsu University, underscoring their recent focus and emerging activity in this field.

### Analysis of authors and journals

Over the past decade, a total of 1,488 authors have made significant contributions to research in this field. We utilized VOSviewer to visualize the network of contributing authors, highlighting both publication volume and collaboration among the top 100 authors (supplementary Fig. S1B). Yu-Qi Feng, Yuting He, and Bi-Feng Yuan have the highest publication volume, each having published five articles in this area. In terms of collaboration, Xiaolan Zhu demonstrates the strongest inclination to work with others, while Yu-Qi Feng and Bi-Feng Yuan exhibit the closest collaborative ties. Consistent with publication volume, the majority of the most prolific authors are based in China, indicating that this field has emerged as a prominent research direction within the country.

**Figure 3:**
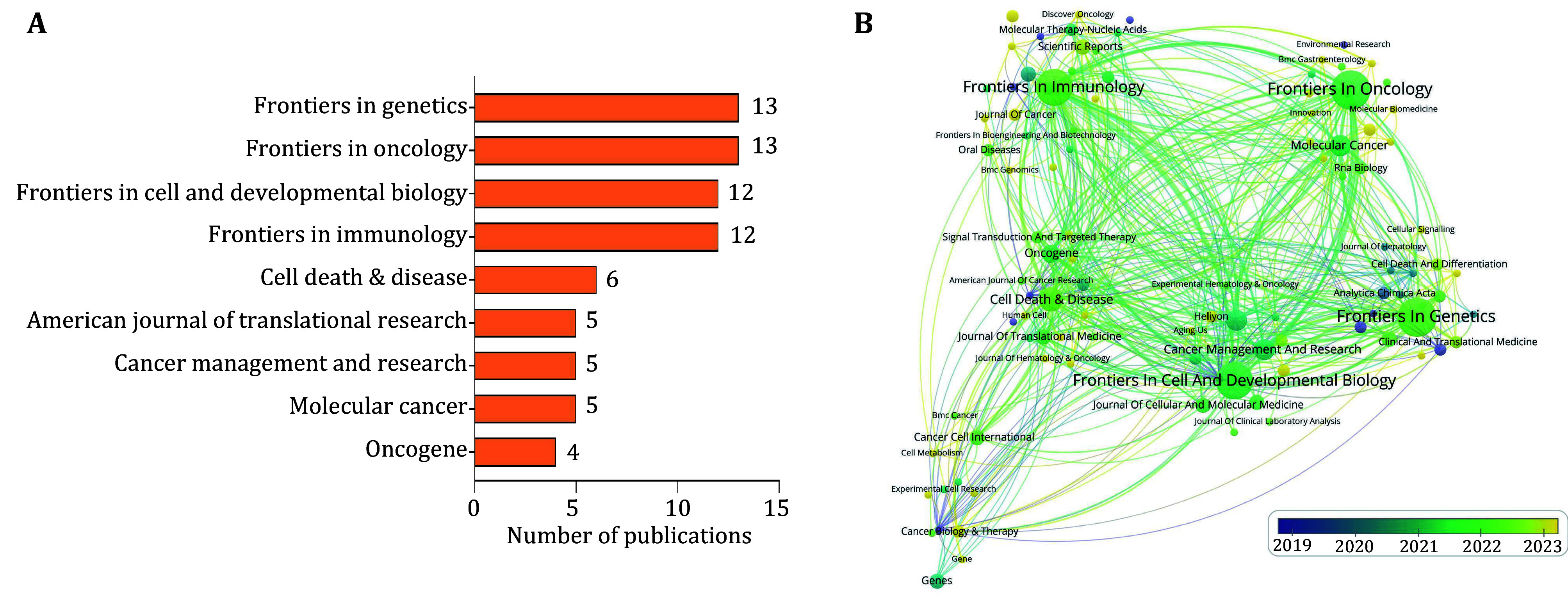
Journal analysis. **A** Number of publications from major journals. **B** The co-occurrence analysis map of journals

A total of 113 journals have published articles on “the relationship between m^5^C methylation modification of RNA and cancer”. The journals with the highest publication counts are *Frontiers in Genetics* (IF_2023_ = 2.8) and *Frontiers in Oncology* (IF_2023_ = 3.5), each featuring 13 articles, followed by *Frontiers in Cell and Developmental Biology* (IF_2023_ = 4.6) and *Frontiers in Immunology* (IF_2023_ = 5.7), each with 12 articles (Fig. 3A). *Frontiers in Genetics* and *Frontiers in Oncology* focus on genetics and cancer research, respectively, aiming to provide researchers with the latest discoveries and breakthroughs in these fields. A clustering analysis of these 113 journals revealed that *Frontiers in Oncology*, *Frontiers in Cell and Developmental Biology*, and *Frontiers in Immunology* exhibited the strongest co-occurrence intensity (supplementary Fig. S1C). In the red cluster, *Frontiers in Immunology* published the most articles; in the green cluster, *Frontiers in Oncology* had the highest article count; in the blue cluster, *Frontiers in Genetics* led with the most articles; in the yellow cluster, *Cell Death and Disease* had the most publications; and in the purple cluster, *Frontiers in Cell and Developmental Biology* produced the highest number of articles (supplementary Fig. S1C). A timeline analysis of publications in these journals indicated that *Molecular BioSystems* and *Biochimie* had earlier publication dates, while the *International Journal of Biological Macromolecules* and *Pathology Research and Practice* are emerging journals in this field (Fig. 3B). This suggests that research on the relationship between m^5^C modification of RNA and cancer has recently become a significant focus for these two journals.

### Analysis of co-cited references

**Figure 4:**
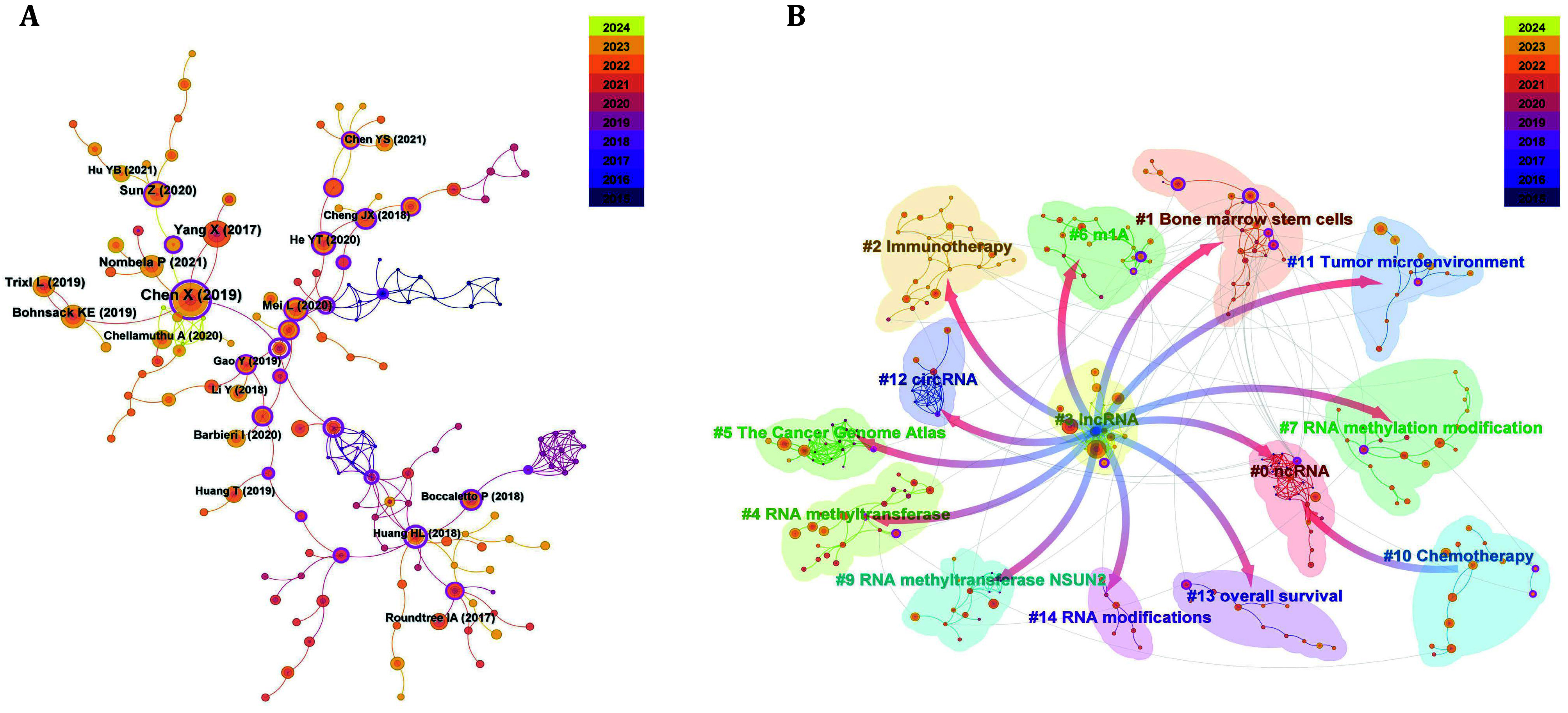
Co-cited references analysis. **A** Co-citied references network map. **B** Co-cited references clustered analysis

Through a CiteSpace analysis, we examined the co-citation of 216 relevant publications spanning from January 2015 to January 2024 (Fig. 4A). The most highly cited paper, authored by Xin Chen *et al*. and titled “5-methylcytosine promotes pathogenesis of bladder cancer through stabilizing mRNAs”(Chen *et al.* 2019), has accumulated 115 citations. This study was published in *Nature Cell Biology*, which has an impact factor of 17.3. The second most cited paper, by Xin Yang *et al*., titled “5-methylcytosine promotes mRNA export: NSUN2 as the methyltransferase and ALYREF as an m^5^C reader”(Yang *et al.* 2017), has garnered 70 citations. This research, published in *Cell Research* with an impact factor of 28.1, elucidated that the RNA methyltransferase NSUN2 catalyzes the formation of m^5^C in mRNA, which is recognized *in vivo* by the mRNA export factor ALYREF. Furthermore, NSUN2 regulates the nuclear-cytoplasmic shuttling of ALYREF, its RNA binding affinity, and mRNA output. The discovery of novel functions of m^5^C methyltransferases and RNA-binding proteins significantly contributes to the advancement of this field of research.

CiteSpace categorized these co-cited publications into 15 clusters: #0 ncRNA, #1 bone marrow stem cells, #2 immunotherapy, #3 lncRNA, #4 RNA methyltransferase, #5 The Cancer Genome Atlas, #6 m^1^A, #7 RNA methylation modification, #9 RNA methyltransferase NSUN2, #10 chemotherapy, #11 tumor microenvironment, #12 circRNA, #13 overall survival, and #14 RNA modifications. Cluster #8 was excluded due to its minimal node count (Fig. 4B). These categories underscore prevalent research interests within this domain, thereby facilitating a systematic and focused review of published articles on specific topics. It is essential to recognize that citation metrics pertain to a defined time frame and may not adequately reflect emerging research frontiers.

### Analysis of keywords

**Figure 5:**
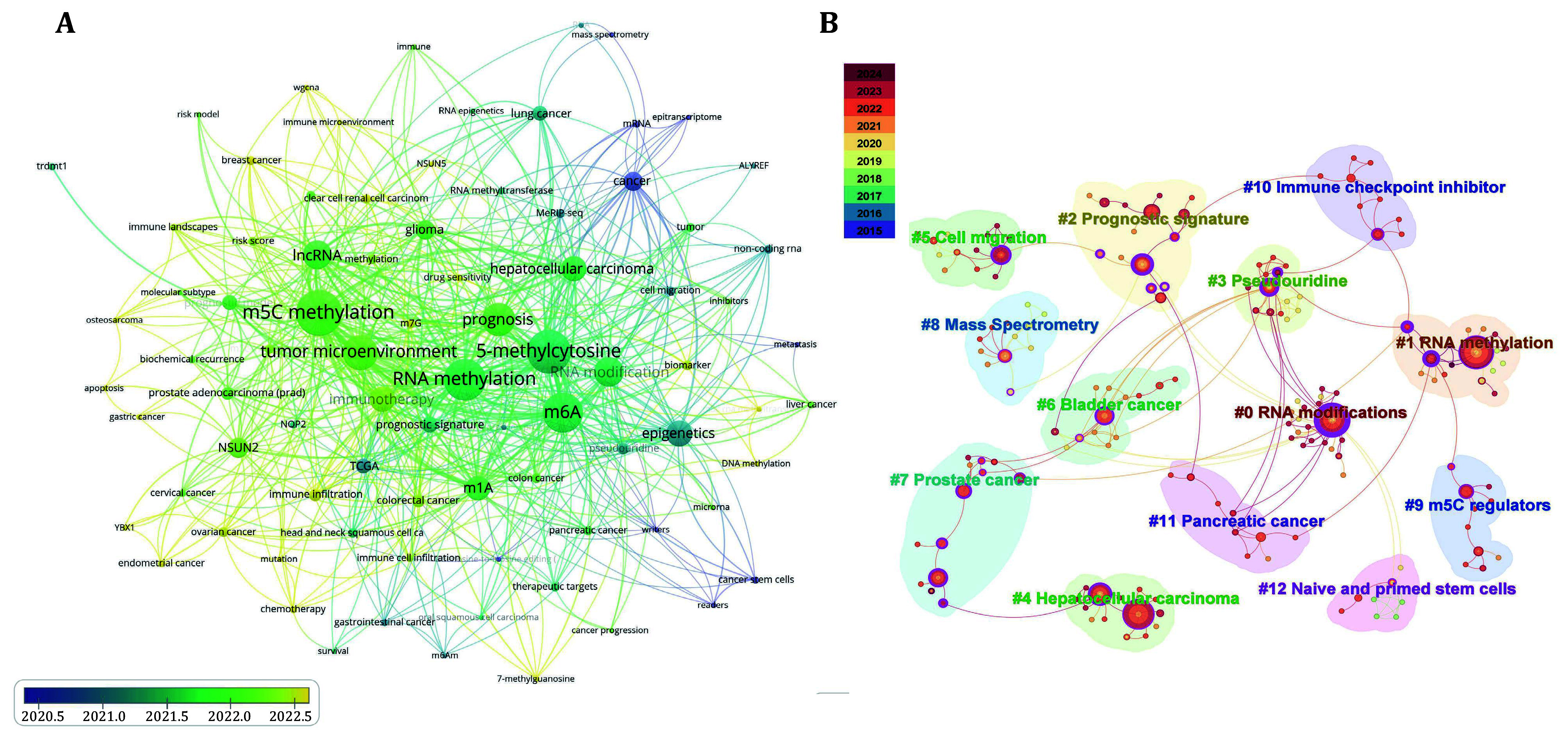
Keywords analysis. **A** Keywords co-occurrence analysis. **B** Keywords clustered analysis

Keywords in academic papers serve as indicators of research hotspots and trends within a specific field over a defined period. Analyzing keyword co-occurrence provides insights into prevailing interests in the scientific domain and suggests future research directions. Utilizing VOSviewer software, we conducted a co-occurrence clustering analysis of keywords from 216 articles, applying a minimum occurrence threshold of two for each keyword. From an initial set of 432 keywords, which was subsequently reduced to 314 after deduplication, we selected 84 keywords for visualization (Fig. 5A). The analysis identified m^5^C methylation (53 occurrences), 5-methylcytosine (49 occurrences), and RNA methylation (45 occurrences) as the most frequently utilized keywords. Additionally, terms such as “readers” and “mass spectrometry” were prominent in earlier stages of research, while “apoptosis” and “chemotherapy” have gained prominence more recently, indicating potential focal points for future research endeavors.

The keyword co-occurrence analysis conducted with CiteSpace categorized these keywords into 13 distinct clusters: #0 RNA modifications, #1 RNA methylation, #2 prognostic signature, #3 pseudouridine, #4 hepatocellular carcinoma (HCC), #5 cell migration, #6 bladder cancer, #7 prostate cancer, #8 mass spectrometry, #9 m5C regulators, #10 immune checkpoint inhibitors, #11 pancreatic cancer, and #12 naive and primed stem cell direction (Fig. 5B).

### Related genes, enrichment analysis and PPI networks

Using VOSviewer software, we performed a co-occurrence clustering analysis of related genes, identifying a total of 1,846 genes. We selected those genes that appeared more than ten times and visualized them based on their functional similarity or co-occurrence in research studies. These related genes were classified into three distinct clusters. Within these clusters, NSUN2 is highlighted in red as the most prominent gene, while AKT1 is represented in blue, indicating significant prominence, and METTL3 is shown in green, also demonstrating notable prominence (supplementary Fig. S1D).

Utilizing the Citexs big data platform, we performed GO and KEGG analyses on 346 genes that were mentioned more than three times in the literature. We employed the R package clusterProfiler to visualize the enrichment analysis of GO terms and KEGG pathways.

**Figure 6:**
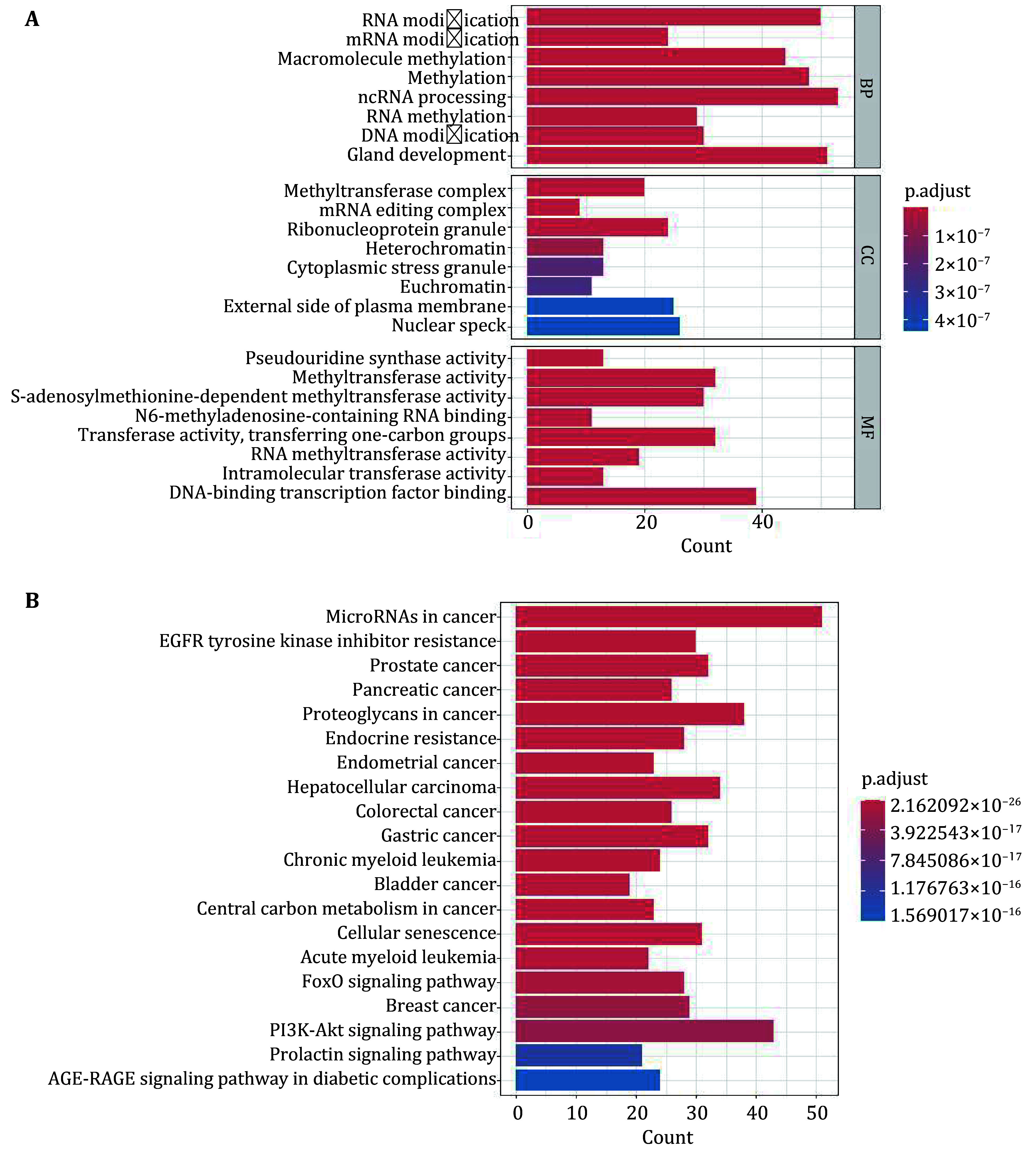
Enrichment analysis of related genes. **A** GO enrichment analysis. **B** KEGG enrichment analysis

GO functional enrichment analysis was conducted with a focus on biological processes (BP), molecular functions (MF), and cellular components (CC) (Fig. 6A). The analysis revealed significant enrichment of genes associated with RNA modification, mRNA modification, and macromolecule methylation within the BP category. In the CC category, notable enrichment was observed in the methyltransferase complex and the mRNA editing complex. For MF, significant enrichment was identified in pseudouridine synthase activity and methyltransferase activity. Additionally, in the KEGG pathway enrichment analysis, the top 20 pathways were selected based on the smallest p-values and highest significance, which were subsequently illustrated in a bar chart (Fig. 6B). The results indicated significant associations with pathways such as microRNAs in cancer, EGFR tyrosine kinase inhibitor resistance, and Prostate cancer, underscoring their relevance in this field of study.

Subsequently, we imported the top 100 ranked proteins mentioned in the article into the STRING database to construct a protein-protein interaction (PPI) network. This network comprised 100 nodes and 675 edges, resulting in an average node degree of 13.5. Using Cytoscape software, we calculated the node degrees and organized them from lowest to highest to create the core protein PPI network graph. The top five proteins identified by degree were TP53, MYC, CTNNB1, HNRNPC, and EGFR, suggesting that these proteins may serve as core components of the network (supplementary Fig. S1E).

## DISCUSSION

M^5^C methylation has been confirmed to play a crucial role in key biological processes including transcription, transport, and translation, which has heightened interest within the academic community. M^5^C methylation is closely associated with the progression of various cancers, such as colorectal cancer, providing empirical support for its significance in tumor biology. Researchers are increasingly focused on understanding how these methylation changes influence biological characteristics, including the proliferation, metastasis, and resistance of cancer cells.

Through this work, we have gained fundamental insights into the impact of RNA m^5^C methylation modifications on cancer biological processes. Utilizing bibliometric research methods, we analyzed the growth patterns, contributions, and collaborations among countries and regions, institutions, and journals, as well as the involvement of prominent researchers and key issues in the field. As a result, we have developed a relatively comprehensive, objective, and scientific understanding of this domain. While there are areas for improvement, this research has also produced notable findings.

### Trends in publication quantity, regional distribution, and journal impact

This study employed bibliometric methods to analyze the growth patterns of research concerning “the relationship between RNA m^5^C methylation modifications and cancer” from 1900 to 2024. We categorized this growth into two stages based on whether the cumulative number of publications over two consecutive years exceeded 10 articles. Prior to 2019, the annual publication volume in this field was fewer than three articles, showing no notable growth trend. However, since 2019, related research has entered a phase of rapid expansion, with the number of publications in both 2022 and 2023 surpassing 60 articles each year. This trend indicates that research on this topic has gradually emerged as a hotspot. Potential factors contributing to this growth include advancements in m^5^C methylation sequencing technology, enhanced academic exchange facilitated by journals, increased funding for research in this area, and the foundational work established by previous studies on the effects of RNA m^5^C modifications on cancer, which have garnered significant interest among researchers. Furthermore, the high mortality rates, challenges in treatment, and severe impacts on human health associated with many cancers ensure that research related to cancer treatment and prognosis remains a consistently prominent topic.

The analysis of publication volumes by countries and institutions indicates that researchers from China have made significant contributions to this field, likely attributable to the availability of advanced experimental systems, sequencing platforms, and analytical methodologies within the country.

The peer review system plays a crucial role in ensuring the rigor and reliability of published scientific research, while the impact factor serves as a commonly utilized metric for evaluating a journal's influence. Between 2015 and 2016, the number of scientific papers in this field was relatively small, resulting in limited impact. For instance, articles published by Elhardt Winfried in *Biochimie* had an impact factor of 3.3, whereas those by Xudong Zhang in *Trends in Molecular Medicine* achieved an impact factor of 12.8 (Elhardt *et al.* 2015; Zhang *et al.* 2016). However, research activity in this field has increased over time. By 2023, articles published in *Cell Metabolism*, *Innovation* (*Cambridge*), and the *Journal of Hematology & Oncology* reported impact factors of 27.7, 33.2, and 29.5, respectively (Chen *et al.* 2023a; Kong *et al.* 2023; Xiong and Zhang 2023). These data indicate a significant improvement in research quality, depth, and breadth, resulting in greater recognition and attention within this field.

Notably, despite the significant contributions of Chinese institutions and researchers, there are relatively few internationally influential academic journals established by Chinese publishers. These journals typically impose stringent requirements regarding the innovation and rigor of research. Consequently, a lack of breakthrough studies may negatively affect the acceptance rate of submissions. Additionally, some research efforts may primarily depend on existing findings, lacking sufficient originality. In the rapidly evolving field of RNA m^5^C methylation, this deficiency in original research could hinder Chinese researchers' ability to compete with their international counterparts. To address these challenges, Chinese research institutions and researchers must prioritize enhancing the quality of their research, fostering international collaboration, and optimizing the evaluation system. By implementing these measures, it is anticipated that China's scientific research outcomes will achieve greater recognition and influence in international journals, thereby securing a more prominent position in global scientific research.

### Highly cited papers discussion

Citation count serves as a measure of the academic impact of a research paper. Highly cited papers often reveal general principles or fundamental themes within a field. By analyzing the co-citation frequencies of these papers, we found that the top 10 most cited papers were published between 2017 and 2021. These papers primarily focused on m^5^C modifications in mRNA and non-coding RNAs such as tRNA, rRNA, and spliceosomal RNA. They investigated the roles of these modifications in cytoplasmic, mitochondrial, and ribosomal assembly and translation, as well as their significance in regulating tRNA and mRNA stability. Key findings include the identification of substrates and targets of m^5^C-related enzymes (reader/writer/eraser), particularly members of the NSUN domain family and YBX1 (Bohnsack *et al.* 2019; Chen *et al.* 2019; Nombela *et al.* 2021; Sun *et al.* 2020b; Yang *et al.* 2017). These studies establish a foundational understanding of the field and are crucial for subsequent research. Research demonstrates that methylation status is intimately connected to the pathogenesis of cancer, encompassing tumor initiation, metastasis, progression, as well as drug resistance and relapse. Numerous methylation regulatory factors show promise as prognostic and diagnostic biomarkers for cancer (Huang *et al.* 2021; Sun *et al.* 2020a). Specific RNA m^5^C modification readers or erasers may serve as potential biomarkers for immune checkpoint blockade, potentially enhancing the effectiveness of cancer immunotherapy (Johnson *et al.* 2003; Lu *et al.* 2020).

In 2017, Yang *et al*. discovered that m^5^C modifications in mRNA are enriched in regions with high CG content and are located downstream of translation initiation sites. These modifications exhibit conserved, tissue-specific, and dynamic characteristics within mammalian transcriptomes. The authors identified NSUN2 as the primary m^5^C methyltransferase responsible for mRNA modifications and demonstrated that ALYREF is a specific mRNA-binding protein essential for facilitating mRNA nuclear export (Yang *et al.* 2017). This study provided valuable theoretical foundations for understanding the biological significance of m^5^C modifications in RNA metabolism, particularly concerning mRNA nuclear export. In 2019, Chen *et al*. presented a nucleotide-resolution map of m^5^C modifications in human urothelial carcinoma of the bladder (UCB). They observed that high m^5^C methylation of oncogenic RNA in UCB correlates with the upregulation of these genes in cancer. The authors identified YBX1 as an RNA m^5^C reader and demonstrated that NSUN2 and YBX1 function as writer and reader, respectively, stabilizing hepatoma-derived growth factor (HDGF) mRNA through m^5^C modification of its 3' UTR, thereby playing a crucial oncogenic role in UCB (Chen *et al.* 2019). This study emphasized that high m^5^C methylation in RNA is a key molecular event in the activation of UCB oncogenes, suggesting that m^5^C modifications could serve as potential targets for cancer treatment and prognosis.

In 2020, Sun *et al*. employed CRISPR/Cas9 to knock down NSUN2 in hepatocellular carcinoma cell lines HepG2 and conducted high-throughput RNA-BisSeq. They demonstrated that NSUN2-mediated m^5^C modification of H19 lncRNA enhances its stability, with m^5^C-modified H19 lncRNA specifically binding to the GTPase activating protein (SH3 domain) binding protein 1 (G3BP1), which in turn leads to MYC accumulation. Furthermore, they compared the expression and m^5^C modification levels of lncRNA H19 in HCC tissues and normal non-cancerous tissues, revealing elevated levels in the cancerous tissues. This study elucidated a novel mechanism by which m^5^C modification of lncRNA H19 exerts its oncogenic effects, thereby providing potential therapeutic targets and biomarkers for the diagnosis and treatment of HCC (Sun *et al.* 2020b).

### Hot topics, key genes and PPI network

Clustering analysis of prominent research topics facilitates a deeper understanding of key areas of study and provides valuable insights for future research directions. The most frequently identified hot topics are “RNA methylation” and “tumor microenvironment”, with occurrences of 45 and 34 times, respectively, thereby shaping current research trajectories. Based on our filtering criteria, which included these two hot topics, we further identified associated cancer types: HCC, prostate adenocarcinoma, lung cancer, colorectal cancer (CRC), and ovarian cancer. These cancer-related keywords collectively underscore critical focal points within this research domain, enhancing our comprehension of the role of RNA modifications in the initiation and progression of cancer.

The temporal frequency map of prominent keywords illustrates the primary periods of their occurrence, thereby improving our understanding of research hotspots throughout various developmental stages in this field. Our findings indicate that the earliest identified hot keyword is “mass spectrometry”, which likely arises from initial research that employed mass spectrometry for the discovery and identification of m^5^C modifications in “readers”. In more recent studies, the keyword “apoptosis” has emerged as particularly prevalent. In 2023, Chen et al. conducted research on cervical cancer, revealing a positive correlation between the expression levels of NSUN2 and LRRC8A. They demonstrated that NSUN2 methylates the anion channel protein LRRC8A, thereby enhancing RNA stability through YBX1 mediation. Additionally, they validated that LRRC8A promotes cancer cell proliferation, migration, and invasion, thereby exerting a pro-tumorigenic role by inhibiting apoptosis. This raises important considerations regarding the relationship between m^5^C modification and apoptosis, as well as their respective roles in the oncogenic process (Chen *et al.* 2023b).

As a prominent contributor to the study of m^5^C modification in RNA, NSUN2 is a key gene frequently examined in significant genetic research, particularly in the context of various cancers. In CRC, NSUN2 facilitates m^5^C modification on the RNA of the SKI-like proto-oncogene SKIL, thereby enhancing the stability of SKIL RNA through YBX1 mediation. NSUN2 acts as a positive regulator of SKIL gene expression, promoting the initiation and progression of CRC tumors (Zou *et al.* 2024). In ovarian cancer, NSUN2 similarly promotes m^5^C modification of E2F1 mRNA, which increases its stability. This regulatory mechanism involves YBX1-dependent modulation of m^5^C modification, establishing a feedback loop between NSUN2 and E2F1. Furthermore, the elevated expression of both NSUN2 and YBX1 in ovarian cancer cells is associated with cancer initiation and progression, correlating with poor patient prognosis (Liu *et al.* 2024b). In HCC, increased NSUN2 expression positively correlates with levels of growth factor receptor-bound protein 2 (GRB2) and HDGF. NSUN2 influences the sensitivity of HCC cells to sorafenib by modulating the Ras signaling pathway, and its depletion results in cell cycle arrest (Song *et al.* 2023). These findings underscore the importance of investigating RNA modifications and their associated writers, readers, and erasers in cancer research, offering valuable insights into cancer therapy and prognosis.

In constructing a Protein–Protein Interaction (PPI) network, we performed both visual and analytical examinations of the top 100 frequently observed protein–protein interactions to elucidate functional associations among proteins related to RNA m^5^C modification and cancer. The analysis identifies the three core proteins within the PPI network: TP53, MYC, and β-catenin (CTNNB1). TP53, also known as tumor protein 53 or p53, functions as a transcription factor and is meticulously regulated by a complex network of post-translational modifications. It exerts various tumor-suppressive functions, including cell cycle arrest, apoptosis, senescence, and autophagy (Liu *et al.* 2024c; Perri *et al.* 2016). MYC, an oncogenic transcription factor encoded by the MYC proto-oncogene, is recognized as one of the most significant drivers of oncogenesis in human cancers. MYC facilitates numerous cancer-associated processes, such as proliferation, self-renewal, survival, genomic instability, metabolism, invasiveness, angiogenesis, and immune evasion, thereby significantly aiding newly transformed cancer cells in evading host immune responses (Dang 2012; Dhanasekaran *et al.* 2022). CTNNB1, a cytoplasmic protein, is involved in various biological processes, including intercellular signaling and the regulation of gene transcription. It has been demonstrated that CTNNB1 promotes the proliferation and invasion of endometrial stromal cells, thus contributing to the progression of endometriosis (Clevers 2006; Shi *et al.* 2024). These essential proteins are critical to the initiation and progression of cancer. Their characterization within the protein interaction network provides valuable insights into the potential mechanisms and functional implications of RNA m^5^C modification in cancer, thereby informing future research endeavors in this field.

### Key genes and virus-related cancer

In the gene analysis list, we identified that in studies concerning RNA m^5^C and cancer, the m^5^C writer NSUN2 is the most frequently mentioned, while YBX1 is the most commonly observed reader. Both genes play a crucial role in the regulation of RNA m^5^C modification across various cancer research studies. A recent study published in May 2024 by Chen *et al*. found that NSUN2 is significantly upregulated in CRC and promotes oncogenesis; YBX1, as a reader, mediates m^5^C-dependent metabolic reprogramming (Chen *et al.* 2024a).

Interestingly, our summary of previous research findings revealed that several viruses, including some oncogenic viruses, are closely associated with these m^5^C writers and readers. The expression level of NSUN2 varies significantly following infection with certain viruses. This alteration may represent a cellular response aimed at regulating viral replication through modulation of NSUN2 expression. The expression level of NSUN2 decreases after infection with various viruses, including SeV, HSV-1, VSV, ZIKV, and SARS-CoV-2 (Wang *et al.* 2023a). In EV71-infected cells, the protein level of the NSUN2 increased after 12 hours post-infection, with its localization shifting from the nucleus to the cytoplasm. In contrast, the levels of other m^5^C-related proteins, including DNMT2, ALYREF, and YBX1, remained unchanged (Liu *et al.* 2024a). Our group found that NSUN2-mediated m^5^C modification increases the stability of HBV RNA and HBV infection significantly upregulates NSUN2 expression in hepatocellular carcinoma cell lines (Feng *et al.* 2023). Li *et al*. discovered that YBX1 recognizes m^5^C modification sites on HCV RNA and enhances the stability of viral RNA, thereby promoting viral replication (Li *et al.* 2024b). In conclusion, RNA m^5^C modification-related writers and readers are closely linked to the development and progression of various virus-induced cancers and play a role in viral genome activities, including stability and replication. This finding provides new insights into the role of m^5^C regulation in cancer progression, suggesting that viral infection may modulate the expression of m^5^C-related genes, thereby influencing cancer development and potentially emerging as a new research focus in the field of RNA modification and cancer.

### Potential implications for developing targeted cancer therapies or biomarkers

The roles of NSUN2 and YBX1 in tumors are critical, indicating that treatment strategies targeting these genes may have potential clinical applications. For instance, inhibitors of NSUN2 could potentially reduce tumor proliferation and migration.

The expression levels of NSUN2 and YBX1 may serve as biomarkers for the early diagnosis and prognosis of cancer. By assessing the expression of these genes, healthcare providers can evaluate patients' cancer risks and prognoses, thereby facilitating the development of personalized treatment plans. Furthermore, treatment strategies that target m^5^C modification-related genes may enhance the effectiveness of existing therapies. For example, combining chemotherapy or targeted therapy with NSUN2 or YBX1 inhibitors may amplify the cytotoxic effects on cancer cells and help overcome treatment resistance.

In summary, NSUN2 and YBX1 play a central role in regulating RNA m^5^C modification, and their interactions significantly influence the occurrence and progression of cancer. These findings not only elucidate new mechanisms in tumor biology but also offer innovative approaches for targeted cancer therapy and biomarker development. Future research should further investigate the specific roles of these genes across various cancer types and explore how to translate these mechanisms into clinical applications, ultimately improving treatment outcomes and survival rates for cancer patients.

## CONCLUSION

In recent years, the relationship between RNA m^5^C modifications and cancer has garnered increasing attention from researchers, leading to a growing number of publications in this area. This study provides a comprehensive summary and analysis of these articles. We have identified the primary research institutions and researchers worldwide who are engaged in this field. Journals such as *Frontiers in Genetics* and *Frontiers in Oncology* are among the most active in disseminating research findings. Xin Chen from Sun Yat-sen University is recognized as the most influential author in this domain. HCC is the cancer type most frequently studied. Key proteins including TP53, MYC, and CTNNB1 have emerged as central elements in these investigations. Our in-depth analysis of related genes suggests that m^5^C-related genes may serve as a link between viruses and virus-related cancers, potentially emerging as a new focus for research. This research offers a detailed overview of current developments and future prospects in this field, aiding researchers in gaining a better understanding of the research background and providing insights for future studies.

## MATERIALS AND METHODS

### Data source and search strategy

The data utilized in this study were sourced from the Web of Science Core Collection database, which is renowned for indexing high-quality academic literature across a wide range of disciplines globally. This database includes significant journals, conference proceedings, patents, and other scholarly publications, establishing it as a widely used bibliometric resource. The study employed the search query TS = (“5-methylcytosine” OR m5C) AND TS=RNA AND TS = (cancer OR malignancy OR neoplasm OR carcinoma OR tumor) to retrieve all publications related to the impact of “RNA m^5^C modification on cancer progression” from January 1, 1900, to January 23, 2024. Subsequent screening involved including articles and reviews relevant to the search terms while excluding letters, brief reports, book reviews, and similar materials, resulting in a selection of 216 highly relevant articles. The selected articles spanned the years 2015 to 2024. This dataset was then utilized for further visual analysis of geographical distribution, institutional affiliations, journal impact, co-cited literature, and keywords pertinent to the research topic. Furthermore, using the Citexs data analysis platform, the study conducted data mining and analysis of genes and diseases referenced in the literature, providing enhanced insights into the genes and diseases closely linked to the exploration of “RNA m^5^C modification and its influence on cancer progression”.

### Data analysis and visualization

In this study, we employed various analytical tools to visualize and analyze data extracted from the Web of Science database. Specifically, we utilized tools such as chorddiag in R, Scimago Graphica, and VOSviewer to create publication analysis graphs categorized by country and region. VOSviewer, a bibliometric visualization tool, was instrumental in generating network visualizations based on authors, keywords, and the co-occurrence of terms, effectively illustrating keyword co-occurrence networks (Jiang *et al.* 2022). Additionally, we employed VOSviewer and Pajek to analyze journal publications, identify the frequency of trending topics, and examine the co-occurrence of genes and diseases. For visual analysis, Citespace was utilized to identify trends in institutional affiliations, citation networks, and the development of keywords, employing complex network algorithms to cluster related nodes and reveal underlying network structures and patterns (Du *et al.* 2024). To conduct functional enrichment analysis of the extracted genes, we used the clusterProfiler, enrichplot, and ggplot2 R packages to visualize Gene Ontology (GO) enrichment and Kyoto Encyclopedia of Genes and Genomes (KEGG) pathway enrichment. A significance threshold of *p* < 0.05 was established to assess the enrichment of GO terms and KEGG pathways within the gene set, with a *p*-value < 0.05 indicating a statistically significant enrichment effect. Furthermore, we constructed and visualized protein–protein interaction (PPI) networks for the extracted proteins using the STRING online platform and Cytoscape. These platforms and software are widely recognized for their utility in bibliometric analysis. Additionally, we conducted statistical analyses of the data using Microsoft Excel 2021, focusing on descriptive statistics, graphical plotting, and curve fitting. We calculated annual publication counts and applied exponential function fitting models, emphasizing the strength of correlation coefficients (*R*^2^). To determine publication growth rates over time, we utilized the following formula: Growth Rate = [(Publications in the previous year / Publications in the first year) / (Last year – First year) – 1] × 100%.

## Conflict of Interests

Authors declare no competing interest from any funding agency.
